# New Role for Cdc14 Phosphatase: Localization to Basal Bodies in the Oomycete *Phytophthora* and Its Evolutionary Coinheritance with Eukaryotic Flagella

**DOI:** 10.1371/journal.pone.0016725

**Published:** 2011-02-14

**Authors:** Audrey M. V. Ah-Fong, Howard S. Judelson

**Affiliations:** Department of Plant Pathology and Microbiology and Center for Plant Cell Biology, University of California Riverside, Riverside, California, United States of America; University of Wisconsin – Madison, United States of America

## Abstract

Cdc14 protein phosphatases are well known for regulating the eukaryotic cell cycle, particularly during mitosis. Here we reveal a distinctly new role for Cdc14 based on studies of the microbial eukaryote *Phytophthora infestans*, the Irish potato famine agent. While Cdc14 is transcribed constitutively in yeast and animal cells, the *P. infestans* ortholog is expressed exclusively in spore stages of the life cycle and not in vegetative hyphae where the bulk of mitosis takes place. PiCdc14 expression is first detected in nuclei at sporulation, and during zoospore formation the protein accumulates at the basal body, which is the site from which flagella develop. The association of PiCdc14 with basal bodies was supported by co-localization studies with the DIP13 basal body protein and flagellar β-tubulin, and by demonstrating the enrichment of PiCdc14 in purified flagella-basal body complexes. Overexpressing PiCdc14 did not cause defects in growth or mitosis in hyphae, but interfered with cytoplasmic partitioning during zoosporogenesis. This cytokinetic defect might relate to its ability to bind microtubules, which was shown using an *in vitro* cosedimentation assay. The use of gene silencing to reveal the precise function of PiCdc14 in flagella is not possible since we showed previously that silencing prevents the formation of the precursor stage, sporangia. Nevertheless, the association of Cdc14 with flagella and basal bodies is consistent with their phylogenetic distribution in eukaryotes, as species that lack the ability to produce flagella generally also lack Cdc14. An ancestral role of Cdc14 in the flagellar stage of eukaryotes is thereby proposed.

## Introduction

Developmental processes are directed by regulatory proteins that coordinate cell division, cellular proliferation, and morphogenesis. While many regulators are conserved over vast evolutionary distances, many have undergone changes in sequence, copy number, or function [1], [2], [3]. Such events have been determinants of biological diversification during the eukaryotic radiation.

One regulator with diverged functions is the dual-specificity protein phosphatase Cdc14. It is best known for its control of mitotic exit in *Saccharomyces cerevisiae*, which involves the antagonism of cyclin-dependent kinases and is regulated by the movement of Cdc14 into and out of the nucleolus during the cell cycle [4]. More recent data indicates that this may not be its main role in most species, however. For instance, the Cdc14-like protein of another yeast, *Schizosaccharomyces pombe*, instead regulates cytokinesis, entry into mitosis, and septum formation [5]. Even in *S. cerevisiae*, evidence is mounting for functions besides mitotic exit, such as in spindle stabilization and DNA replication [6]. In metazoans such as *Caenorhabditis elegans*, humans, mouse, and *Xenopus laevis*, many roles in the cell cycle are described including in cytokinesis, G1/S and G2/M transitions, meiosis, and/or DNA damage checkpoints, especially in vertebrates which contain two or more Cdc14 genes [7], [8], [9], [10], [11]. Cdc14 of *C. elegans* also helps program lineage-specific mitotic blocks during development [12] and a mouse Cdc14 regulates oocyte maturation [13].

Despite many studies of Cdc14, a unified view of its cellular function or ancestral role has not emerged. One limitation of current knowledge is that most research has targeted species in adjoining phylogenetic clades, in the Fungi/Metazoa group. The known activities of Cdc14 may therefore not reflect its origins, or the full diversity of its functions. In this study, we expand our understanding of Cdc14 through studies of *Phytophthora infestans*, the potato late blight agent [14]. *P. infestans* has a fungus-like growth habit, but lacks taxonomic affinity with true fungi; it is classified as an oomycete, and belongs to the Kingdom Stramenopila along with diatoms and brown algae [15]. *P. infestans* is an interesting system for studying Cdc14 since it does not exhibit a classic cell cycle, instead forming coenocytic hyphae in which nuclei divide asynchronously [16].

In a prior study, we reported that the expression pattern of the single Cdc14 gene of *P. infestans*, *PiCdc14*, differs strikingly from that of known homologs [17]. Instead of being regulated post-translationally like its fungal and metazoan relatives, *PiCdc14* is under strong transcriptional control with its mRNA produced only when hyphae begin to make asexual sporangia. The absence of mRNA from vegetative hyphae is due to a lack of transcription rather than instability [18]. *PiCdc14* transcripts persist in sporangia, which are metabolically active but mitotically quiescent, and in the zoospores that are released from sporangia. Zoosporogenesis is stimulated by cool conditions and involves the rapid cleavage of the cytoplasm of each multinucleate sporangium into six or more mononucleate zoospores, each with two flagella anchored at basal bodies, in a process that has some resemblance to cytokinesis in other eukaryotes [19]. *PiCdc14* mRNA disappears after zoospores encyst and form germ tubes, in which mitosis resumes. Our prior data did not indicate if PiCdc14 protein also accumulated in spores, but gene silencing blocked sporulation [17].

Here we further address the role of *PiCdc14* in *P. infestans* and report a novel activity. PiCdc14 accumulated in nuclei during early sporulation, based on the use of fusions with green fluorescent protein (GFP). During zoosporogenesis PiCdc14 became a prominent component of basal bodies, which has not been described previously in any species. PiCdc14 also interacted with microtubules *in vitro*, and overexpression caused abnormal cleavage of sporangial cytoplasm during zoosporogenesis. Combined with our discovery of a strong evolutionary linkage between Cdc14 and flagella, this suggests that an ancestral role of Cdc14 may be in basal bodies or other aspects of the development of flagellated cells.

## Results

### Localization of PiCdc14 during *P. infestans* development

N- and C-terminal fusions were constructed between PiCdc14 and GFP and named GFP/PiCdc14 and PiCdc14/GFP, respectively. Similar fusions from other species retained their cellular activity and distribution [20]. Nevertheless, before expressing GFP/PiCdc14 and PiCdc14/GFP in *P. infestans*, whether they would complement a *cdc14^ts^* mutation in *S. cerevisiae* was tested. We showed previously that PiCdc14 complements this mutation [17], and GFP/PiCdc14 and PiCdc14/GFP were found to function similarly.

*P. infestans* transformants expressing GFP/PiCdc14 or PiCdc14/GFP behind the native *PiCdc14* promoter were then generated. Both showed similar patterns of expression, which matched expectations from prior RNA blot and promoter studies of the native gene [17], [18]. Nonsporulating cultures lacked fluorescence, but expression was observed in young sporulating cultures, sporangiophores, sporangia, zoospores, and zoospore cysts. Levels of the PiCdc14 fusion proteins declined as cysts germinated. Details are presented next, based on observations of paraformaldehyde-fixed tissues.

**Figure 1:**
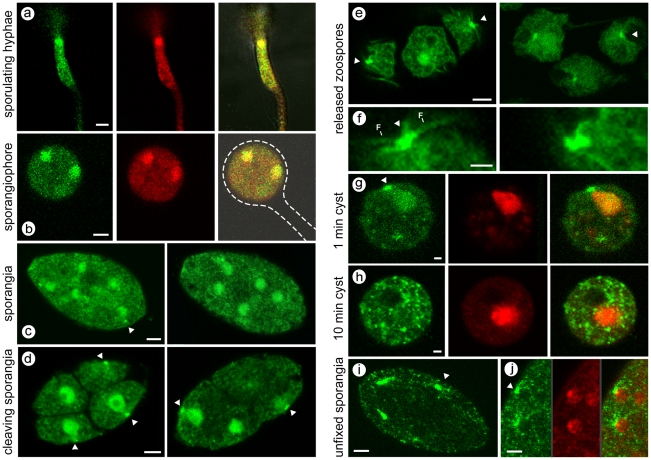
Transformants expressing PiCdc14/GFP under control of native promoter. (**A**) Hyphae in culture induced to sporulate, with GFP channel (left), red DRAQ5 channel (center), and merged images (right) showing that much PiCdc14/GFP resides in the nuclei. (**B**) Same as prior row except illustrating a young sporangiophore, with the sporangiophore and immature sporangium outlined. (**C**) Two freshly harvested sporangia, with PiCdc14/GFP concentrated in nuclei and “dots”, indicated by arrow. (**D**) Cleaving sporangia, with PiCdc14/GFP in peripheral dots indicated by arrows. (**E, F**) Zoospores with staining in putative basal bodies, with the latter shown at higher magnification with flagella marked in the lower panel. (**G, H**) Zoospores fixed 1 and 10 min after inducing encystment, showing GFP, DRAQ5, and merged channels. (**I, J**) Unfixed sporangia, which can be compared with the paraformaldehyde-fixed material in the other panels; in panel J the GFP, DRAQ5, and merged channels are shown to stress the absence of PiCdc14/GFP from nuclei. Bars equal 4 µm in all panels except *F–H*, where they equal 1 µm.

In young sporulating cultures, PiCdc14 was first detected in short regions of hyphae (Figure 1A). This was not seen in nonsporulating hyphae, *i.e.* young or submerged cultures, so likely represent the initial sites of sporangiophore development. Much of the signal colocalized with nuclei based on DRAQ5 staining. As sporulation proceeded, PiCdc14 entered sporangiophores and then sporangia; this is illustrated in Figure 1B, where most PiCdc14 has moved into the sporangium, which has not yet swelled to its final ovoid shape or formed its basal septum. PiCdc14 shows a clear nuclear signal, and a weaker punctate cytoplasmic signal.

A similar distribution of PiCdc14 was observed in mature sporangia, except that bright dots or specks were occasionally seen adjacent to nuclei, typically facing the sporangial wall (Figure 1C). The dots became more numerous and pronounced when sporangia were chilled to initiate zoosporogenesis (Figure 1D). Nuclei at this stage have acquired a pyriform shape with their peaks oriented conspicuously towards the PiCdc14 dots. Prior studies have shown that this site, near the narrow ends of the nuclei, is the location of the two basal bodies [21].

Zoospores in the swimming stage showed a similar distribution of PiCdc14 (Figure 1E). PiCdc14 dots were adjacent to the cell surface, at the presumptive basal bodies near the ventral groove. These frequently had an elongated appearance or were manifested as two closely spaced dots, which suggested that Cdc14 associated with each basal body (Figure 1F). Some PiCdc14 was throughout the cell, including flagella.

Zoospore encystment involves flagella detachment and cell wall formation, and this was linked with the elimination of PiCdc14. One minute after inducing encystment by vortexing, PiCdc14 was still seen at both presumptive basal bodies and the nucleus (Figure 1G). After 10 min, PiCdc14 dispersed throughout the cytoplasm (Figure 1H). During this the nuclei returned to a round profile, and PiCdc14 fluorescence was eventually lost.

The above patterns were observed in multiple transformants expressing either the N- and C-terminal GFP fusions. The distribution was also the same in transformants expressing a phosphatase-dead version of PiCdc14. In human cells and *X. laevis*, catalytic activity is also not required for Cdc14 localization to centrosomes [9].

While Cdc14 has been localized to nucleoli, centrosomes, or spindle pole body in yeasts and animal cells, Cdc14 was never observed at those sites in *P. infestans* in any developmental stage. For example, Figure 1D shows that Cdc14 is largely non-nucleolar during zoosporogenesis. It should be noted, however, that unlike the situation in yeasts and animals, Cdc14 is not expressed during the mitotically active stages (*i.e.* hyphae) of *P. infestans*. Most nuclear division ceases at an early stage of sporangiophore development [22]. Sporangia become multinucleate as a result of the migration of several nuclei into each sporangium, and not by division in the developing sporangium [23].

### Localization of PiCdc14 in unfixed spores

Compared to the observations listed above for fixed tissues, slightly different results were obtained from unfixed samples. While PiCdc14 dots were still near the periphery of unfixed cleaving sporangia, no nuclear fluorescence was detected (Figure 1I, J). For example, PiCdc14 can be seen in dots outside, but not within, nuclei in Figure 1J. This suggests that the affinity of PiCdc14 for nuclei is transient compared to its more durable interaction with the presumptive basal bodies. The latter is consistent with data shown later that suggest some PiCdc14 aggregates with cytoskeletal components.

While fixation can yield artifacts, it is suggested that the fixed samples described in the previous section (which show a greater degree of nuclear localization) may better represent the real-case situation for ungerminated sporangia. This is because unfixed sporangia, when placed under the microscope at room temperature, start to undergo cytoplasmic changes associated with direct germination, a process that entails the formation of a hyphal germ tube instead of zoospores. This involves reorganization of the cytoplasm and elimination of flagellar proteins in addition to PiCdc14.

### Presence of PiCdc14 in flagella-basal body complexes (FBBC)

**Figure 2:**
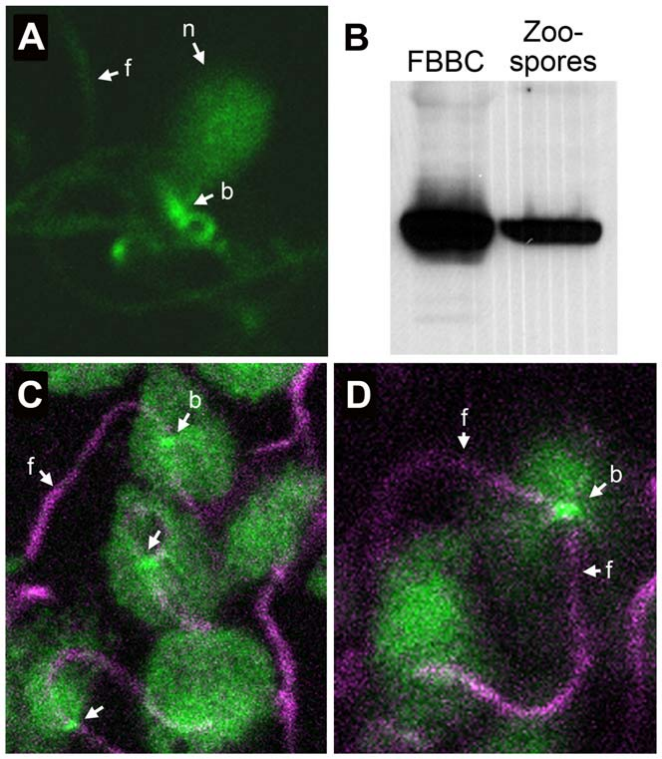
PiCdc14 association with flagellar basal body complexes. (**A**) FBBC from strain expressing PiCdc14/GFP, showing the protein in basal bodies (b), flagella (f), and nuclei (n). (**B**) Detection of PiCdc14/StrepTag in purified FBBCs and whole zoospores, using equal amounts of protein per lane and anti-StrepTag. (**C, D**) Colocalization in zoospores of PiCdc14/GFP (green) with flagella (pink, stained with anti-β-tubulin). Basal bodies and selected flagella are indicated. Bars represent 2 µm.

To help confirm the association of PiCdc14 with basal bodies, FBBCs were isolated from zoospores by a detergent-based method used with *Chlamydomonas* [24]. The resulting complexes contain flagella, two basal bodies, fibers between the basal bodies, and attached nuclei. When FBBCs were isolated from PiCdc14/GFP-expressing transformants, microscopic analysis showed that the fusion protein accumulated at the basal bodies, at flagellar roots near the tip of pyriform nuclei (Figure 2A).

The concentration of PiCdc14 in the FBBC was demonstrated further by studying transformants expressing PiCdc14 fused to the 30-aa Strep-Tag. Western blot analysis using Strep-Tag antibody indicated that PiCdc14 was at least 5-fold enriched in FBBCs compared to total zoospore proteins (Figure 2B).

### Colocalization of PiCdc14 with β-tubulin and DIP13

Additional information about subcellular location was obtained by labeling PiCdc14/GFP zoospores with anti-β-tubulin, which detects flagella. As shown in Figure 2C and 2D, flagella (pink signals) emerge from the points of PiCdc14/GFP accumulation at the presumptive basal bodies.

**Figure 3:**
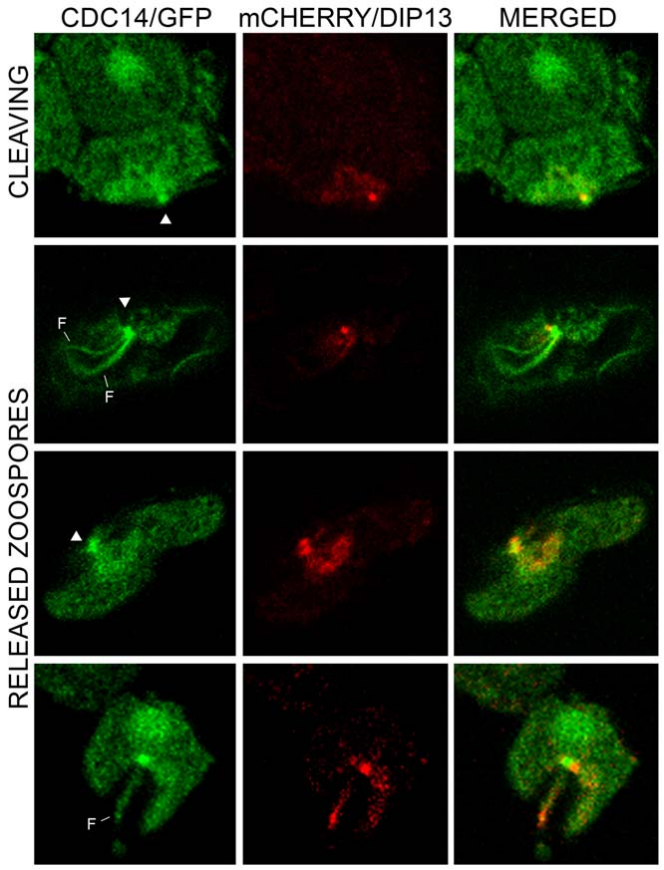
Colocalization of PiCdc14 and DIP13. Shown are the locations of the two proteins in transformants expressing Cdc14 and DIP3 fused to GFP or mCherry, respectively, in a cleaving sporangium (top row) and zoospores (bottom rows). Indicated are the basal bodies (arrowheads) and flagella (F). Bars represent 4 µm.

Further support for the association of PiCdc14 with the basal body came from colocalization studies with DIP13. PiDIP13 has 65% amino acid identity with its *Chlamydomonas* homolog, which is a known basal body marker [25]. In transformants coexpressing mCherry/PiDIP13 and PiCdc14/GFP, both labeled structures near the site of flagella attachment, but the signals were slightly offset (Figure 3). Often two adjacent specks were seen for PiDIP13, corresponding to each basal body. While the precise location of DIP13 in the basal body of other species is unknown, this suggests that PiCdc14 resides near the edge of each basal body.

### PiCdc14 binds microtubules *in vitro* and forms insoluble complexes *in vivo*

**Figure 4:**
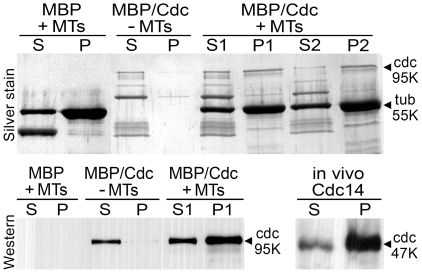
Complex formation by PiCdc14. Top panel: silver-stained gel from a microtubule binding assay, in which PiCdc14 fused to MBP and StrepTag (MBP/Cdc) or MBP alone from *E. coli* were incubated with or without microtubules (MT). After centrifugation, pellets (P) and supernatants (S) were resolved by SDS-PAGE and stained to detect the 95 kDa PiCdc14 fusion band. The strong 55 kDa band is tubulin, and the strong lower band in the left-most lane is MBP. Lanes S1/P1 and S2/P2 represent samples from independent experiments. A blank lane was deleted at the site marked by a vertical line. Lower panels: Western blots probed with anti-StrepTag. The lower left image shows samples from the upper gel, and confirms that PiCdc14 binds microtubules *in vitro*. The bottom right blot shows the partitioning of PiCdc14/StrepTag protein from *P. infestans* between supernatant (S) and pellet (P), and suggests that most PiCdc14 is insoluble *in vivo*.

Other data indicated the *P. infestans* protein can bind microtubules, like other Cdc14 proteins [7], [11]. This was demonstrated in co-sedimentation assays in which taxol-stabilized microtubules were mixed with recombinant PiCdc14 containing maltose binding protein (MBP) at its N-terminus and a C-terminal Strep-Tag (Figure 4, top panel). The majority of the 95 kDa MBP/PiCdc14 fusion pelleted when mixed with microtubules. Densitometry indicated that 65% of MBP/PiCdc14 pelleted in one experiment (lane P1 vs. S1) and 76% in one performed on a separate day (P2 vs. S2), compared to 6% in controls lacking microtubules. The fusion protein appeared unstable during the assay, possibly due to proteases, resulting in bands smaller than 95 kDa. Western analysis using antibodies to the Strep-Tag confirmed that the 95 kDa band co-sedimenting with the microtubules is PiCdc14 and not a contaminant (Figure 4, bottom left).

The above result, coupled with the localization data, prompted us to investigate whether native PiCdc14 is partially insoluble in *P. infestans*, which would be expected for a microtubule or basal body-associated protein. When proteins from a *P. infestans* transformant expressing PiCdc14/StrepTag were centrifuged at 14,000×*g*, over 80% of PiCdc14 was in the pellet based on Western analysis using antibody to the tag (Figure 4, bottom right). Similar results were obtained in *Chlamydomonas* with DIP13 and other basal body proteins [26].

### Effects of PiCdc14 overexpression

PiCdc14 can release the defect in mitotic exit caused by *cdc14^ts^* in *S. cerevisiae*, yet is not expressed in the mitotic cells (*i.e.* hyphae) of *P. infestans* [17]. It was thus of interest to test the effect of expressing PiCdc14 in hyphae. Transformants were made that expressed PiCdc14/StrepTag or PiCdc14/GFP behind the strong constitutive *Ham34* promoter. Analyses of the transformants indicated that the cellular levels of the tagged versions of Cdc14 ranged from being about equal to that of native Cdc14 in sporulating hyphae to five times higher.

**Figure 5:**
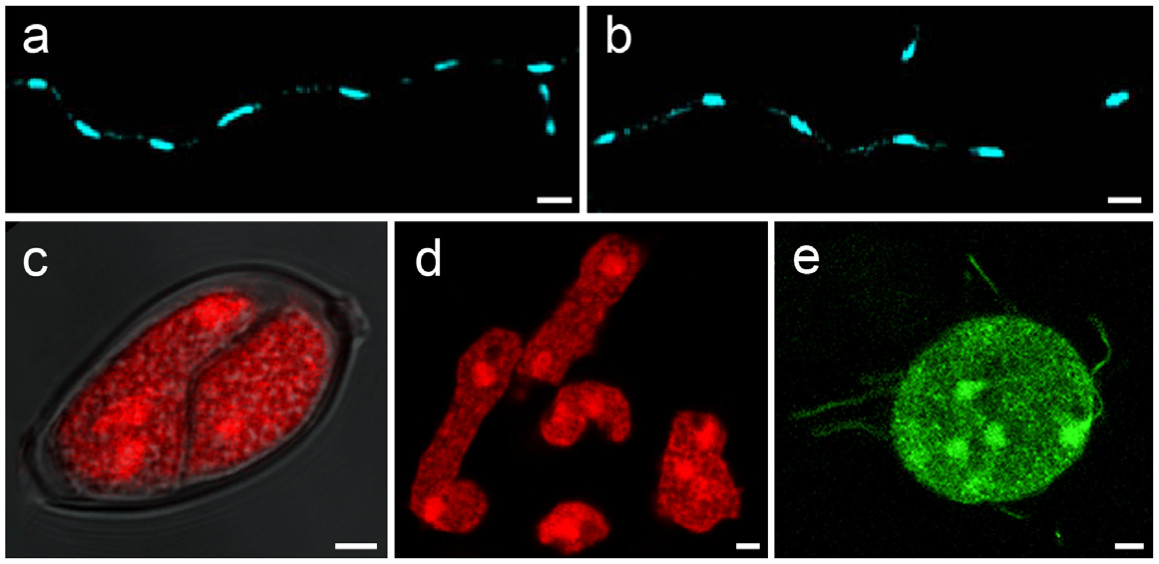
Expression of PiCdc14/GFP using a strong constitutive promoter. Top panels show typical DAPI-stained hyphae from wild type (**A**) and Ham34-PiCdc14/GFP transformants (**B**), which appear similar. Lower panels show abnormal cleavage patterns, including missing cleavage planes in sporangia, with DRAQ5 highlighting multinuclearity (**C**); DRAQ5-stained multinucleate zoospores (**D**); and a GFP image of an uncleaved “superzoospore” (**E**). These can be compared to normal patterns in Figure 1, where Cdc14 was expressed behind a weaker and spore-specific promoter. Bars denote 4 µm.

Both the PiCdc14/StrepTag and PiCdc14/GFP transformants exhibited normal nuclear behavior, growth, and sporulation, and their spores were able to resume nuclear division after germination. As shown in Figure 5 for hyphae, the size and distribution of nuclei in controls and transformants were similar. Since overexpression of Cdc14 causes cellular abnormalities in other systems [7], [9], [27], [28], [29], [30], this suggests that PiCdc14 does not affect mitosis in *P. infestans*, at least in hyphae. In addition, nuclei size, number, and the timing of division appeared similar in the sporangiophores and sporangia of the overexpressing strains. Compared to hyphae in which mitosis is asynchronous, nuclear division is synchronous in sporulating tissues [23].

The use of gene silencing to test whether PiCdc14 has a direct role in zoospore or flagella function is not possible since silencing prevents the formation of sporangia [17]. However, a role of PiCdc14 in zoospore formation was revealed by overexpression studies, which caused major defects in zoosporogenesis. As shown in Figure 1, zoospores normally have a single nucleus. However, most zoospores from the overexpressing strains were multinucleate. It was common to observe incomplete cleavage occurring within sporangia (Figure 5C; compare with Figure 1), zoospores with 2–3 nuclei (Figure 5D), and multiflagellated “superzoospores” reflecting a total lack of cleavage (Figure 5E). These abnormalities increased with the level of PiCdc14 expression. For example, in a transformant expressing PiCdc14/GFP at low levels based on the intensity of GFP fluorescence, 12±4% of zoospores were multinucleate, and 29±13% in a strain with high levels. By comparison, an average of 1.0±0.7% of zoospores were multinucleate in wild type controls, and 1.4±0.8% in transformants expressing GFP alone. The number of abnormal zoospores in strains overexpressing Cdc14/StrepTag also increased with expression level, ranging from 10 to 73%. Whether this occurs as a consequence of its interaction with microtubules or interference with phosphorylation/dephosphorylation events is yet to be determined. Nevertheless, this indicates that while PiCdc14 may not function as a mitotic regulator in hyphae, it might play a role in cytokinesis during cleavage.

### Evolutionary linkage between Cdc14 and flagella

Our finding that PiCdc14 associates with basal bodies and flagella led to speculation that organisms lacking these structures would also lack Cdc14. A search of the sequenced genomes of 22 species selected to represent the nine commonly accepted eukaryotic kingdoms supported the hypothesis (Table 1). Most notably, no Cdc14-like sequences were detected in *H. arabidopsidis*, an oomycete related to *P. infestans* that is unable to make zoospores. Cdc14 is likewise absent from higher plants, which lack flagellated life-stages, but present in lower plants with flagellated stages such as the moss *P. patens* and the green algae *C. reinhardtii*, a chlorophyte. Significantly, both Cdc14 and flagella are lost from another chlorophyte, *O. tauri*.

**Table 1:** Distribution of Cdc14 and flagella-associated structures among eukaryotes.

Classification		Species	Basal bodies	Flagella or cilia	Cdc14	Centrioles
Alveolata	Ciliophora	*Tetrahymena thermophilia*	+	+	+	+
"	Apicomplexa	*Toxoplasma gondii*	+	+	+	+
Amoebozoa	Eumycetozoa	*Dictyostelium discoideum*	−	−	−	−
"	Archamoebae	*Entamoeba histolytica*	−	−	−	−
Animalia	Nematoda	*Caenorhabditis elegans*	+	+	+	+
"	Chordata	*Homo sapiens*	+	+	+	+
"	Chordata	*Xenopus laevis*	+	+	+	+
Euglenozoa	Kinetoplastida	*Trypanosoma brucei*	+	+	+	−
Excavata	Percolozoa	*Naegleria gruberi*	+	+	+	−
Fungi	Chytridiomycota	*Batrachochytrium dendrobatidis*	+	+	+	+
"	Ascomycota	*Saccharomyces cerevisiae*	−	−	+	−
Plantae	Bryophyta	*Physcomitrella patens*	+	+	+	+
"	Chlorophyta	*Ostreococcus tauri*	−	−	−	−
"	Chlorophyta	*Chlamydomonas reinhardtii*	+	+	+	+
"	Lycopodiophyta	*Selaginella moellendorffi*	+	+	+	+
"	Streptophyta	*Arabidopsis thaliana*	−	−	−	−
Rhodophyta	Bangiophyceae	*Cyanidioschyzon merolae*	−	−	+	−
Stramenopila	Oomycota	*Phytophthora infestans*	+	+	+	+
"p	Oomycota	*Hyaloperonospora arabidopsidis*	−	−	−	+
"p	Oomycota	*Pythium ultimum*	+	+	+	+
"p	Oomycota	*Saprolegnia parasitica*	+	+	+	+
"	Bacillariophyta	*Thalassiosira pseudonana*	+	+	+	+

Cdc14 sequences were identified from public databases and validated by the reciprocal best Blast strategy. Centrioles includes structures with either standard triple or singlet tubules [32], [43]. Although *H. arabidopsidis* has not been examined for centrioles, their presence is inferred based on other downy mildews [44].

Two exceptions exist to the association between Cdc14 and flagella. These are the red algae *C. merolae* and *S. cerevisiae*, which contain Cdc14 but lack flagella. Other Cdc14-containing fungi such as chytrids have flagella, however.

**Figure 6:**
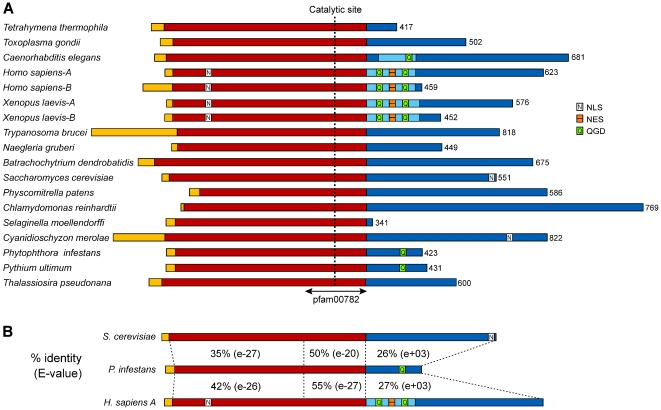
Structures of Cdc14 proteins. (**A**) Proteins from the species in Table 1. The sequences are taken from their respective genome databases, except for the *Naegleria*, *Selaginella*, *Trypanosoma*, and *Thalassiosira* proteins which are based on manually curated gene models. The predicted proteins range from 341 to 822-aa as marked to the right of each model. Following a N-terminal region that shows little similarity between the proteins (yellow), each protein contains a fairly conserved stretch of about 300 aa (red). The latter includes the phosphatase domain which is marked as pfam00782, with the catalytic residue indicated. The C-terminal portions of the proteins (blue) show little conservation except for a roughly 85 aa region that is fairly conserved between *C. elegans*, human, and *X. laevis* (light blue). This includes the nuclear exit sequence (NES) and one or two QGD repeats. Nuclear localization signals (NLS) are also marked as detected by PSORTII; these include an experimentally validated NLS near the C-terminus of the *S. cerevisiae* protein [45], NLSs in the N-terminal regions of the human and *X. laevis* proteins which appear to have functions based on mutagenesis studies [20], [46], and a NLS predicted in the C-terminal region of the *C. merolae* protein. (**B**) Similarity between Cdc14 of *P. infestans*, *S. cerevisiae*, and human Cdc14A. The program SSEARCH was used to calculate the percent amino acid identity in the region upstream, upstream, and C-terminal to the pfam00782 phosphatase domain. *E*-values for each match are also provided, which indicate that the similarity at the C-terminus is insignificant.

Comparisons of Cdc14 sequences taken from the species in Table 1 provide minor insight into the evolution of Cdc14 function. As shown in Figure 6A, Cdc14 proteins contain a usually short N-terminal region that shows little similarity between species, a well-conserved central region of about 320-aa that includes the dual specificity phosphatase motif, and a highly variable C-terminal region [7]. PiCdc14 fits this paradigm with a short 15-aa N-terminal extension, a 316-aa conserved central region, and an 85-aa C-terminus. The *P. infestans* protein is roughly equidistant between human and budding yeast Cdc14, averaging 39, 53, and 27% identity upstream, within, and downstream of the phosphatase domain (Figure 6B). By comparison, *S. cerevisiae* Cdc14 and human Cdc14A show 33, 50, and 18% identity in these regions, respectively.

**Figure 7:**
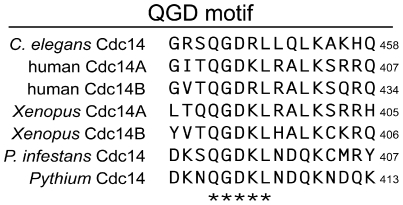
Conserved QGD motif in the C-terminal region of Cdc14. Illustrated are alignments of the region containing the motif, indicating that QGDKL is conserved within each oomycete and metazoan Cdc14. Numbers at the right indicate the position within each protein, and conserved residues are marked at the bottom of the alignment.

PiCdc14 lacks an canonical nuclear localization signal (NLS), unlike some metazoan and *S. cerevisiae* proteins which contain these at their N- and C-termini, respectively. A survey of proteins from the kingdoms listed in Table 1 indicated that most also lack a canonical NLS, which suggests functional divergence during evolution. All stramenopile Cdc14s group strongly with each other but weakly with other groups in phylogenetic analyses (not shown), providing little insight into which type of Cdc14 they evolved from. However, a QGD-containing motif of unknown function first noted in *C. elegans*, human, and *X. laevis* [20] is present within PiCdc14 as well as orthologs from other oomycetes including *Pythium* (Figure 7). It is not possible to test this motif for function in *P. infestans* since gene replacements can not be performed due to a lack of homologous recombination and diploidy, but it is notable that the QGD motif exists only in the oomycete and metazoan orthologs.

## Discussion

The original discovery of Cdc14 as a regulator of mitosis in the *S. cerevisiae* cell cycle led scientists to search for similar roles in other fungi and in animals. This resulted in a recognition that Cdc14 activities are more diverse than first thought, but maintained a focus on its role in cell cycle progression [7]. Our work with *P. infestans*, which has a different evolutionary history than the prior-studied models, has revealed new roles of Cdc14, and raised questions about its ancestral function and how eukaryotes evolved. Persuasive evidence for the new roles include our findings that Cdc14 localizes to basal bodies in *P. infestans*, and that flagella and Cdc14 have been generally coinherited during eukaryotic evolution.

The evolutionary argument for the linkage of Cdc14 with flagella is particularly compelling when considering groups where some members coordinately lost both features (*i.e. Hyaloperonospora* versus *Phytophthora* in the Oomycota, and *Ostreococcus* versus *Chlamydomonas* in the Chlorophyta). If Cdc14 was essential for mitosis, it should have been retained in these phyla after the loss of flagella. That Cdc14 is not required universally for mitosis is evident by its absence from higher plants, which also lack centrioles. The argument that Cdc14 is required for mitosis in organisms that use centrioles is weakened by the fact that hCdc14A or hCdc14B are not absolutely essential for cell cycle progression in human cell lines [31].

Relationships between flagella and mitotic regulators should not be surprising. Basal bodies are microtubule organizing centers for flagella, while centrioles are microtubule organizing centers for the mitotic spindle. Basal bodies and centrioles are structurally related and interconvert during development in most species [32]. The accumulation of PiCdc14 at the basal body of *P. infestans* thus parallels findings of its homologs at human and frog centrosomes, and the yeast spindle pole body [7]. Whether flagella-anchoring basal bodies or centrioles involved in mitosis appeared first during evolution has been debated, but one theory is that flagella evolved first as a motility and sensory organelle, and the basal body was later co-opted into a mitotic role [33], [34], [35].

This leads to our proposal that an ancestral role of Cdc14 was to regulate the function or biogenesis of the flagellar apparatus, an activity that has been maintained in *P. infestans*. PiCdc14 may also serve to inhibit mitosis in the motile stage, or transform centrioles into basal bodies, which may also anchor regulatory proteins besides Cdc14 [36]. In the common ancestor of animals and fungi, Cdc14 may have adapted to a function in mitosis in addition to its role in the flagellated stage. In certain lineages that lost flagella during evolution, such as *S. cerevisiae* and *S. pombe*, Cdc14 may have been retained to serve regulatory roles during mitosis or cytokinesis. Groups with other mitotic mechanisms, such as higher plants which lack centrioles, could afford to lose the protein.

A role of PiCdc14 in cytokinesis during zoosporogenesis, *i.e.* cleavage, may also exist in light of its ability to bind microtubules. Certain basal body proteins, as well as some Cdc14s, are known to interact with microtubules [7], [11], [25], [37]. This might serve several purposes in *P. infestans* including anchoring the microtubule rootlet to basal bodies, or stabilizing the flagellar rootlet during cytokinesis. Our data do not discriminate between binding to microtubules at the basal body or elsewhere, but an association with microtubules driving cleavage [38] is consistent with the defects seen in transformants overexpressing PiCdc14. Overexpression of PiCdc14 may misregulate proteins through excessive dephosphorylation or sequestration of its substrates. Misexpression similarly interferes with cytokinesis in many species [28], [29], [30].

Our overexpression experiments, combined with our earlier analysis of *PiCdc14* transcription and the timing of accumulation of PiCdc14 protein revealed in this study, suggest that PiCdc14 does not regulate mitosis during normal growth. This differs strongly from the situation described in other eukaryotes [4], [9], [27], [29], [30]. PiCdc14 could nevertheless still be capable of interacting with its traditional mitotic substrates in *P. infestans*. Genes encoding homologs of traditional yeast and animal substrates such as cyclin, Polo kinase (Cdc5), and Wee kinase are detected in the *P. infestans* genome; that these might be affected by PiCdc14 is suggested by our finding that the latter can complement a defective version of *cdc14* in *S. cerevisiae* [17]. However, such traditional targets of Cdc14 might not be expressed or accessible to the phosphatase during hyphal growth in *P. infestans*. This may change during sporulation, which may also explain why sporulation is blocked by silencing PiCdc14 [17]. During sporulation PiCdc14 may control nuclear behavior or monitor novel developmental checkpoints, in addition to its later role in basal bodies.

Finally, we predict that the presence of Cdc14 in the basal body is not unique to oomycetes. The lack of prior reports of its association with cilia or flagella probably reflects the absence of those structures from the model systems (yeasts and animal somatic cell lines) that were employed in most studies.

## Materials and Methods

### Developmental stages of *P. infestans*

All experiments were performed using isolate 1306, using cultures grown in the dark at 18°C. Nonsporulating hyphae were obtained from 3-day rye-sucrose broth cultures inoculated with 10^4^ sporangia/ml. Liquid cultures grown for 5 days (sporulation begins after 3–4 days) were the source of sporulating hyphae. For zoospore production, sporangia were collected from 8-day rye-sucrose agar cultures by flooding the plates with water, rubbing with a glass rod, and separating sporangia from hyphal fragments by passage through 50-µm nylon mesh. Indirect germination (*i.e.* zoosporogenesis) was induced by placing a sporangial suspension (10^5^/ml) on ice for about 20 min, followed by incubation at 10°C. Cleaving sporangia, *i.e.* sporangia that were in the process of differentiating zoospores but had not yet released zoospores, were obtained after about 30 min of incubation at 10°C. Free-swimming zoospores were obtained after 60 min of additional of incubation at 10°C, and purified from sporangia by passage through 15-µm mesh. To stimulate encystment, zoospore suspensions were adjusted to 0.5 mM CaCl_2_ and vortexed for 30 sec. Germinating cysts were obtained by incubating the cysts in water at 10°C for 12 h.

### *P. infestans* transformation

Stable transformants were obtained by treating protoplasts with plasmids using G418 selection [39]. The plasmids were constructed starting from pSAM or pTOR, which were kindly provided by F. Mauch. Inserts for these were generated by polymerase chain reaction as described below, adding restriction sites as needed.

The PiCdc14/GFP construct containing the sporulation-specific native promoter, *i.e.* expressing GFP fused to the C-terminus of PiCdc14, included the PiCdc14 ORF (open reading frame) and 945 bp of upstream DNA. Prior studies had demonstrated that this promoter region conferred the native pattern of expression [18]. The promoter-ORF fragment was amplified from genomic DNA and inserted into the *EcoR*I and *Not*I sites of pSAM, in front of GFP which was followed by *Ham34* transcriptional terminator [40].

The GFP/PiCdc14 construct was constructed using pTOR as a backbone, by first inserting the Cdc14 promoter into its *Kpn*I-*Cla*I sites, then inserting GFP taken from pSAM into *Eco*RV-*Not*I sites, and finally placing the Cdc14 ORF into the *Not*I site. The resulting construct thus expresses the GFP/Cdc14 fusion using the native promoter and the *Ham34* terminator. For constitutive expression, the PiCdc14-GFP-Ham34 terminator fragment from the PiCdc14/GFP plasmid was inserted in the *EcoR*I and *Xba*I sites of pTOR, behind the constitutive *Ham34* promoter of *Bremia lactucae* [40].

Phosphatase-dead constructs were made by mutating Cys277 to Ser in the catalytic site. This was generated from the PiCdc14/GFP plasmid by site-directed mutagenesis using the QuikChange Site-Directed Mutagenesis kit from Stratagene.

The PiCdc14/StrepTag plasmid was made by fusing the Strep-tag, also known as One-STrEP-Tag [41], to the C-terminus of the PiCdc14 ORF. This was done by ligating double-stranded oligonucleotides designed to encode SAWSHPQFEKGGGSGGGSGGGSWSHPQFEK using optimal codon usage for *P. infestans* to the ORF. The fusion was then joined to its native promoter and subcloned in pTOR.

The mCherry/PiDIP13 plasmid was made by subcloning the fluorescent tag from pmCherry (Clonetech) into the *Cla*I-*Spe*I sites of pTOR. The *Ham34* promoter in pTOR was then replaced with a 977 bp promoter from PiDIP13 (gene model PITG_13461 in the *P. infestans* database at the Broad Institute of Harvard and MIT) using *Kpn*I-*Cla*I sites just upstream of mCherry, and then the PiDIP13 ORF was inserted downstream of mCherry using *Spe*I and *Sac*II.

### Lysates and flagella-basal body complexes

Whole cell extracts were made from liquid nitrogen-ground sporulating hyphae resuspended in 50 mM Tris pH 6.8, 5 mM EDTA, 10% v/v glycerol, and protease inhibitor cocktail, and clarified by spinning at 4°C for 10 min at 14,000×*g*. FBBCs were isolated by an adaptation of a published method using MT buffer [24]. This entailed mixing zoospores suspended in MT with an equal volume of MT plus 2% Triton X-100 and protease inhibitors. After stirring 10 min on ice, this was layered above 50% Percoll in MT, centrifuged at 14,500×*g* for 30 min, and then FBBCs recovered from the interface were diluted in MT, washed twice, and resuspended in MT. Immunoblots of FBBCs or extracts were prepared by transferring proteins from SDS-PAGE to nitrocellulose, incubation with StrepTag antibodies (IBA), and visualization using the ECL system (GE Healthcare).

### Microscopy

Samples were prepared according to Hardham [42]. Fixation was performed for 30 min in 4% paraformaldehyde, 50 mM PIPES pH 6.8. Tissues were then pelleted for 5 min at 1000×*g*, washed twice for 5 min each at room temperature in 100 mM PIPES buffer, once in PBS (20 mM sodium phosphate, 150 mM NaCl, pH 7.4), and resuspended in water at 10^5^ cells/ml. Samples were mounted using Vectashield (Vector Laboratories) as an antifade agent. DNA staining was performed using 20 µM DRAQ5 (Biostatus Ltd.).

For immunomicroscopy, fixed samples (15-µl aliquots) were applied to 8-well glass slides (Nunc) in an equal volume of 0.2% Triton X-100 in 100 mM PIPES pH 6.8. After 30 min, the wells were rinsed in water, air-dried for 45 min at 37°C, rehydrated in PBS (20 mM sodium phosphate, 150 mM NaCl, pH 7.4), and incubated for 60 min at 37°C with rabbit polyclonal anti-β-tubulin (Abcam, diluted 1∶200 in PBS, 1% BSA). Cells were then washed three times for 5 min in PBS, incubated with Alexa Fluor 633-labelled goat anti-rabbit IgG for 60 min at 37°C (Invitrogen, diluted 1∶750 in PBS, 1% BSA), and then rinsed three times in PBS and once in water.

Imaging was performed using a laser-scanning confocal Zeiss LSM510 using 63× water or 100× oil immersion objectives and the manufacturer's settings for the desired wavelengths. Initial image analyses were performed using Zeiss LSM Image Browser software, and later Adobe Photoshop was used to adjust image brightness and generate overlays.

### Recombinant protein production

Coding sequences were inserted into pMAL-c2x (New England Biolabs) and expressed in *E. coli* BL21. Cultures in 2×YT were induced with 0.3 mM IPTG for 2 h at 37°C, sonicated, and the fusion protein was purified on amylose resin.

### Microtubule binding assay

Input proteins included MBP alone, or a fusion of MBP, PiCdc14, and Strep-Tag. These were expressed in *E. coli*, purified using amylose columns, and exchanged into 80 mM PIPES pH 7.0, 1 mM EGTA, 1 mM MgCl_2_ using a desalting column just prior to the assay. Microtubule binding was measured using the Microtubule Protein Spin-Down Biochem Assay Kit (Cytoskeleton, Inc.) as directed by the manufacturer, using a 100,000×*g* centrifugation step. The resulting supernatants and pellets were resolved by SDS-PAGE and silver-stained.

### Yeast transformation

PiCdc14/GFP and GFP/PiCdc14 were tested for their abilities to complement *cdc14^ts^* of *S. cerevisiae* as described [17]. This involved expressing the ORFs behind the GAL1 promoter and testing transformants for growth at 25°C and non- 37°C in the presence and absence of galactose.
